# Stress, coping strategies, and relapse among schizophrenia patients at the psychiatric hospital of Oujda, Morocco

**DOI:** 10.11604/pamj.2025.52.97.44612

**Published:** 2025-11-04

**Authors:** Salah-Eddine El Jabiry, Mohammed Amine Bouazzaoui, Mohammed Barrimi, Fatima Elghazouani, Bouchra Oneib

**Affiliations:** 1 Department of Psychiatry, Mohammed VI University Hospital of Oujda, Oujda, Morocco,; 2 Faculty of Medicine and Pharmacy, Mohammed First University of Oujda, Oujda, Morocco,; 3 Maternal-Child and Mental Health Research Laboratory, Faculty of Medicine and Pharmacy of Oujda, Mohammed First University, Oujda 60000, Morocco,; 4 Epidemiology, Clinical Research and Public Health Laboratory, Faculty of Medicine and Pharmacy of Oujda, Mohammed First University, Oujda 60000, Morocco

**Keywords:** Schizophrenia, relapse, stress, coping, psychiatric care

## Abstract

**Introduction:**

schizophrenic relapse constitutes a formidable challenge in the realm of psychiatric care, often precipitating a cascade of deteriorating symptoms and functional impairment. Addressing this issue necessitates a comprehensive understanding of the factors that contribute to relapse occurrence.

**Methods:**

this cross-sectional study, conducted at the Hospital of Mental Health and Psychiatric Diseases in Oujda, sought to explore the intricate interplay between stress, coping mechanisms, and the incidence of relapse among individuals diagnosed with schizophrenia. Over six months, data were collected from three hundred schizophrenia patients, encompassing demographic, clinical, stress, and coping-related variables.

**Results:**

analysis of the findings unveiled compelling associations between stress intensity, coping efficacy, and relapse vulnerability. Notably, heightened stress intensity emerged as a significant predictor of relapse occurrence, surpassing the mere exposure to stressors. Furthermore, inadequate coping strategies were found to significantly elevate the risk of relapse, underscoring the pivotal role of adaptive coping skills in mitigating relapse susceptibility. In addition to these associations, the study revealed intriguing insights into the demographic and clinical characteristics of the patient population. Predominantly male and urban-dwelling, the patients exhibited varying levels of education and employment status. A substantial proportion reported a history of substance abuse and psychiatric family history, indicating potential predisposing factors for relapse vulnerability. These findings underscore the imperative of integrating stress management interventions and fostering adaptive coping mechanisms within schizophrenia treatment paradigms. Early identification and targeted interventions aimed at ameliorating stress reactivity and enhancing coping proficiency. Also, it held promise for the reduction of relapse rates and the increase of long-term outcomes for individuals grappling with schizophrenia.

**Conclusion:**

by elucidating the nuanced dynamics of stress and coping in relation to relapse, this study contributes valuable insights to the development of tailored interventions and therapeutic strategies in schizophrenia management.

## Introduction

Schizophrenic relapse is a frequent occurrence. It is commonly described as the “re-emergence or worsening of psychotic symptoms” (i.e., delusions and hallucinations) [1,2]. It is considered that 35% of schizophrenic patients present a significant number of relapses, and thus experience a progressive and marked deterioration in the course of their pathology [3]. Moreover, more than half of all patients relapse within two years of their first episode [4].

There is a consensus on the clinical importance of relapse episodes in schizophrenia. Their frequency, nature, and intensity have a prognostic value, as they are factors that adversely affect the course of the disorder. Several factors are implicated in the relapse phenomenon, including stress. Stress has been defined as a non-specific adaptive response to a stressor, comprising three phases: the alarm reaction, the resistance phase, and the exhaustion phase. The identification of neuroendocrine reactions linked to stress has led to extensive research into the relationship between stressful events and the onset of disease [5]. Some authors have suggested that living with schizophrenia is like “living in a minefield” and that, at the slightest stress, the illness reappears [6]. The term “coping” refers to the cognitive and behavioral efforts made by an individual to adapt to the demands of his environment when these exceed his immediate capabilities. This process aims to regain internal balance, either by using personal resources or by resorting to the resources offered by the social environment [7]. Lazarus *et al*. cognitive theory of stress-coping helps to understand the use and function of adaptive strategies in the face of stressors [8].

Several authors have reported that people with schizophrenia, who demonstrate good stress management skills and coping strategies, are at lower risk of psychotic decompensation [9]. To achieve this objective, the present investigation will scrutinize patients' demographic as well as clinical attributes who experience relapses within the schizophrenic spectrum. Moreover, an evaluation will be conducted to discern the influence exerted by stressors and stress in addition to the efficacy of coping mechanisms on the recurrence of schizophrenic episodes.

## Methods

**Study design:** this is a cross-sectional study to evaluate the relationship between stress, coping strategies, and the incidence of relapse among individuals diagnosed with schizophrenia.

**Study setting and population:** this study was spread over a period of six months, from November 2020 to April 2021, at the Hospital of Mental Health and Psychiatric Diseases at CHU Mohammed VI.

**Inclusion criteria:** patients over 16 years of age, who had already relapsed and became stable or at least in partial remission (reporting an improvement in the signs of schizophrenia without total disappearance), were included to be followed up in an outpatient clinic with a medical record before the start of the study.

**Exclusion criteria:** patients in decompensation were excluded, along with those who refused to take part in the study. Information was collected from the questions that were asked to the patient, his family, and the medical record.

**Variables:** the variables studied in our present study mainly consist of a dependent variable, which is the schizophrenic relapse, and several independent variables, which are: age, sex, marital status, origin, socio-economic level, educational level, psychiatric, medical, and surgical history, stress, and coping modalities.

### Data resource and measurement

**Data source:** the data was merged into a database using the software SPSS Inc.^©^ Statistical Package for the Social Sciences (version 21), and stored on a password-protected computer.

**Sources of measurements:** two instruments were used to assess stress and coping.

**Brief stress and stressor evaluation scale (Cungi 1997):** the self-assessment scale for stress and stressors [10] is translated and validated in French: i) the questionnaire comprises 11 items to explore 11 groups of possible reactions resulting from stress. The subject rates the importance of the reaction, from one very little reaction to six extremely important reactions. The score ranges from 11 to 66. ii) The questionnaire consists of eight items exploring eight groups of potential stressors. The subject rates the importance of each item, from one with very little impact to six extremely important impacts. The score ranges from eight to 48.

**Brief COPE:** the French-Canadian version of the Brief COPE [11] (a 28-item self-report questionnaire) is used to measure coping strategies for stressful everyday events. The Brief COPE has 14 scales (self-distraction, active coping, denial, substance, emotional support, instrumental support, behavioral disengagement, ventilation, positive reframing, planning, humor, acceptance, religion, and self-blame). Each of these scales comprises two items measuring different coping strategies, some of which are considered adaptive and others more problematic and less adapted. Responses are calculated on a Likert-type scale ranging from ''not at all'' [1] to ''always'' [4].

**Bias:** guarantee of participant anonymity to reduce reporting bias. We have used validated scales. Implement appropriate statistical techniques to control potential biases in the analysis of data.

**Sample size:** we made an exhaustive recruitment of all respondents.

**Statistical analysis:** the statistical analysis was carried out with SPSS Inc. software^©^ Statistical Package for the Social Sciences (version 21) by the epidemiology and clinical research department of the Faculty of Medicine. The statistical analysis took place in two descriptive and analytical stages.

**Descriptive study:** qualitative variables were described in terms of numbers and proportions, and quantitative variables were described depending on normality in terms of mean and standard deviation or median and interquartile range.

**Analytical study:** after verifying the normality of the number of relapses variable, a bivariate analysis was performed using the non-parametric Wilcoxon-Mann-Whitney test for the purpose of comparing ranks. Hence, a p<0.05 was considered significant.

**Ethical consideration:** data collection was carried out with respect for the anonymity of the patients and the confidentiality of their information throughout the different times of the study. The study protocol was approved by the Ethics Committee of the Faculty of Medicine and Pharmacy of Oujda (Version: December 2020).

## Results

**Epidemiological data:** three hundred patients were recruited. The mean age of the patients was 38.04 ± 11.42, with extremes ranging from 17 to 70. The majority (85.7%) were male; most of the patients recruited (81.3%) lived in urban areas. Most of the patients recruited (77.7%) were single, as most patients in our sample attended school (90.7%). More than half (56.3%) of the patients in our sample had no professional activity. Over half the patients (59.7%) lived with their parents, whereas only 3.3% lived alone.

**Personal and family history:** a larger part of our patients (89.7%) had no personal medical or surgical history, but the majority (72.3%) in our sample had a history of substance abuse. Almost half (49%) had a psychiatric family history.

**Clinical data:** the extremes of schizophrenic relapses range from 1 to 21 relapses, with an average number of 4.49 over the course of the disease. The mean number of relapses per year was 0.47 ± 0.42, with extremes ranging from 0.03 to 2.33 relapses per year. Forty-eight percent (48%) of patients had a high-intensity stressor with a median of 4 and IQ [2; 7], while 45.7% of patients had a high-intensity stressor with a median of 5 and IQ [3; 7.5]. Most patients (69.3%) achieved inadequate coping with stress, with a median of 6 and an IQ [4; 8] (Table 1). This section analyzed the influence of stress and coping on the occurrence of schizophrenic relapse. On the one hand, high level of stressor was not associated with a high risk of relapse (p=0.28), and on the other hand, high stress intensity was correlated with an increased risk of relapse (p<0.001), in addition to an adapted coping, which was associated with a higher risk of relapse with a p<0.001 (Table 2).

**Table 1:** numbers and percentages of the different variables studied

Variable	Workforce	Percentage (%)
**Gender**		
Male	257	85.7
Female	43	14.3
**Origin**		
Urban	244	81.3
Rural	56	19.7
**Marital status**		
Single	233	77.7
Married	53	17.7
Divorced	14	4.6
**Schooling**		
Yes	272	90.7
No	28	9.3
**Professional activity**		
Yes	169	56.3
No	131	43.7
**Personal medical and surgical history**		
Yes	34	11.3
No	266	89.7
**Family psychiatric history**		
Yes	147	49
No	153	51
**SPA consumption**		
Yes	217	72.3
No	83	27.7
**Stressor**		
Low	157	52
High	143	48
**Stress**		
Low	163	54.3
High	137	45.7
**Coping**		
Adapted	51	17
Unadapted	115	38

**Table 2:** association between stress and coping with schizophrenia relapse

Variable	Median+ IQ	P-value
**Stressor** (Cungi)		0.28
Low	4 [2; 5]	
High	4 [2; 7]	
**Stress** (Cungi)		<0.001
Low	3[2; 5]	
High	5[3;7.5]	
**Coping**		<0.001
Adapted	2 [1; 3]	
Unadapted	6 [4; 8]	

## Discussion

Schizophrenic relapse remains a problem in the course of the illness. In this study, we sought to assess the impact of stress and coping skills on schizophrenic relapse in our sample. High stress intensity and inadequate coping would be associated with an increased risk of relapse in these patients. According to the vulnerability-stress model, when a person with schizophrenia is confronted with different environmental stressors, the likelihood of exacerbation of psychotic symptomatology and decompensation is increased [12,13]. The greater the vulnerabilities a person presents, the less stress is required to trigger a psychotic episode [14]. Several studies have demonstrated the presence of environmental stressors in the period preceding psychotic relapse [15-17]. More specifically, several researchers note the presence of stressful life events around three weeks before psychotic decompensation [18-20]. These stressors can be both internally related to cognitions and emotions, and externally, such as job loss. These events are considered triggers for the psychotic relapse process [21]. Simonet *et al*. argue that stressors disrupt the homeostasis of people with schizophrenia [22]. Performance pressure at work or at school can lead to the exacerbation of psychotic symptoms [22]. It is the management of the reaction to anxiety-provoking situations that often leads to frank decompensation or not [21]. Reducing these stressors (removing the anxiogenic stimulus) or acquiring stress management skills should decrease the likelihood of decompensation [12].

In our study, exposure to high-intensity stressors was not associated with a risk of schizophrenic relapse, whereas high-intensity stress was linked to a risk of schizophrenic relapse. As claimed by the vulnerability-stress model, coping skills act as a moderator in the individual's response to stressful stimuli [14]. The term “coping” refers to the cognitive and behavioral efforts made by an individual to adapt to the demands of his environment when these exceed his immediate capabilities. This process aims to regain internal balance, either by using personal resources or by resorting to the resources offered by the social environment [8]. There are two main types of coping strategies: problem-solving and emotion-focused. Emotion-focused coping is less adaptive since it emphasizes the avoidance of painful emotions, but it is sometimes necessary when situations become uncontrollable [23]. Both the general population and people with severe mental disorders use the same range of coping strategies to manage psychosocial stressors [24]. Gingerich point out that coping skills can reduce the impact of stress on psychotic symptomatology and lower the risk of decompensation [9]. When a person can adapt to new events, stress, and symptom management and reduction are enhanced [23]. The literature on coping strategies used by forensic psychiatric patients is sparse [23]. In our study, inadequate coping skills in the face of stress are associated with the risk of schizophrenic relapse.

## Conclusion

Schizophrenia is a chronic psychiatric illness with a major influence on patients' quality of life. It is characterized by numerous relapses, aggravating the illness over time. These relapses are linked to several factors, including stress. As our study shows, stress and poor stress management are associated with the risk of relapse. It is important, thus, to measure as early as possible, preferably from the first psychotic episode, to prevent the patient from entering a chronic phase marked by numerous relapses, progressive worsening of the illness, and diminished response to antipsychotic treatment.

### What is known about this topic

- Several factors contribute to schizophrenia's relapse;
- Good stress management skills and coping strategies are at lower risk of psychotic decompensation.

### What this study adds

- High stress intensity and inadequate coping would be associated with an increased risk of relapse in patients with schizophrenia in the Moroccan context.
